# Fucoidan from *Ericaria crinita* Alleviates Inflammation in Rat Paw Edema, Downregulates Pro-Inflammatory Cytokine Levels, and Shows Antioxidant Activity

**DOI:** 10.3390/biomedicines11092511

**Published:** 2023-09-11

**Authors:** Paolina Lukova, Elisaveta Apostolova, Alexandra Baldzhieva, Marianna Murdjeva, Vesela Kokova

**Affiliations:** 1 Department of Pharmacognosy and Pharmaceutical Chemistry, Faculty of Pharmacy, Medical University-Plovdiv, 4002 Plovdiv, Bulgaria; 2 Department of Pharmacology, Toxicology, and Pharmacotherapy, Faculty of Pharmacy, Medical University-Plovdiv, Vasil Aprilov Str. 15A, 4002 Plovdiv, Bulgaria; 3 Department of Medical Microbiology and Immunology “Prof. Dr. Elissay Yanev”, Faculty of Pharmacy, Medical University-Plovdiv, Vasil Aprilov Str. 15A, 4002 Plovdiv, Bulgaria; 4 Research Institute at Medical University-Plovdiv, Vasil Aprilov Str. 15A, 4002 Plovdiv, Bulgaria

**Keywords:** fucoidan, *Ericaria crinita*, *Cystoseira crinita*, anti-inflammatory effect, rat paw edema, carrageenan, cytokines, antioxidant activity

## Abstract

Fucoidans are sulfated polysaccharides detected mainly in the cell walls of brown seaweeds. Here, we examined the effects of single doses of fucoidan derived from *Ericaria crinita* (formerly *Cystoseira crinita*) on carrageenan-induced paw inflammation in rats. The serum levels of TNF-α, IL-1β, IL-6, and IL-10 of rats with LPS-induced systemic inflammation after 14 days of treatment were also evaluated. Subchronic treatment with fucoidan from *E. crinita* attenuated the inflammation during the late phase of the degraded carrageenan-induced paw edema (3rd to 5th hour after carrageenan injection) with peak activity at the 3rd hour after the application. Both doses of fucoidan from *E. crinita* (25 and 50 mg/kg bw) significantly decreased the levels of all tested pro-inflammatory cytokines (IL-1β, TNF-α, and IL-6) in the serum of rats with a model of system inflammation but had no effect on the anti-inflammatory cytokine IL-10. The results showed that the repeated application of fucoidan has a more prominent effect on the levels of some pro-inflammatory cytokines in serum in comparison to a single dose of the sulfated polysaccharide. This reveals the potential of *E. crinita* fucoidan as an anti-inflammatory agent. Furthermore, *E. crinita* fucoidan exhibited *in vitro* antioxidant capacity, determined by 2,2-diphenyl-1-picryl-hydrazyl radical scavenging and ferric reducing antioxidant power assays as follows: IC_50_ = 412 µg/mL and 118.72 μM Trolox equivalent/g, respectively.

## 1. Introduction

The inflammatory response to potentially harmful stimuli (injury, stress, or infections) includes the release of pro-inflammatory mediators such as tumor necrosis factor-α (TNF-α), interleukin-1β (IL-1β), and interleukin 6 (IL-6). TNF-α induces macrophage activation and increases the production of IL-1β, IL-6, and prostaglandin E2. IL-6 is known as an endogenous mediator of lipopolysaccharide (LPS)-induced fever. IL-1β is produced mainly from macrophages and is involved in the pathophysiology of rheumatoid arthritis. The elevated levels of these substances promote prolonged inflammation and further infiltration with monocytes, granulocytes, lymphocytes, and mast cells at the site of injury. When activated, these cells could promote excessive inflammation (manifested with pain and edema) and subsequent tissue damage [1,2,3,4]. 

Reactive oxygen species (ROS) are synthesized by the body as a result of the normal metabolism of the cells. The small or moderate quantities of ROS in the organism play physiological roles such as in signaling processes and defense mechanisms against infectious agents, while high concentrations may disrupt the equilibrium of the pro-oxidants/antioxidants in the body and lead to oxidative stress [5,6]. The high concentrations of ROS can cause lipid, protein, and DNA damage and contribute to many pathological conditions, including cardiovascular and respiratory diseases, neurological disorders, cancer, diabetes, etc. The human body possesses antioxidant systems (enzymatic and non-enzymatic) which are capable of neutralizing free radicals in physiological conditions, though in a state of disturbed homeostasis, they may not be sufficient to protect the body effectively. Therefore, the use of exogenous antioxidants has been considered the most efficient approach to diminish oxidative stress [6,7,8]. 

However, none of the commercial antioxidant and anti-inflammatory agents exhibit ideal characteristics. At this point, there is a continuing search for new substances that can adjust to the changing conditions [9]. Therefore, a number of natural products, including seaweeds, are widely investigated. Marine macroalgae are classified as green (*Chlorophyta,* Chlorophyceae), brown (*Ochrophyta,* Phaeophyceae), and red (*Rhodophyta*, Rhodophyceae) macroalgae. Brown algae synthesize anti-inflammatory and antioxidant compounds such as phlorotannins, carotenoids, tocopherol, and fucoxanthin. These substances persist in a low percentage of algae mass; moreover, their production is associated with some economic and ecological problems [10]. Nonetheless, the polysaccharides fucoidan, alginate, and laminarin can represent 50–70% of the dry weight of some brown seaweeds [11]. Furthermore, they are non-toxic and water-soluble and their isolation process is less harmful to the environment [9,12].

Fucoidans are sulfated polysaccharides detected often in the cell walls of brown algae. The chemical structures and compositions of fucoidans vary significantly depending on the geographical location, species, seasons, and population age [4,13]. However, generally, they have a skeleton of repeating α-(1→3) linked L-fucopyranose residues or alternating α-(1→3) and α-(1→4) linked L-fucopyranoses, substituted with sulfate and/or acetate groups. Fucoidans may have several other sugar molecules such as glucose, arabinose, xylose, galactose, and uronic acid. Multiple research articles focus on the diverse pharmacological activities of fucoidans, including anticancer, immunomodulatory, antiviral, antimicrobial, antidiabetic, antioxidant, anti-inflammatory, and anticoagulant activities [4,14,15]. 

*Ericaria crinita* (*E. crinita*) (formerly *Cystoseira crinita*) is a brown macroalgae with wide distribution on the Mediterranean Sea and the Black Sea waterfronts. Our recent study reported the results from a chemical composition and structure analysis of fucoidan isolated from *E. crinita.* Moreover, we have reported that single doses of this polysaccharide exhibited a well-defined anti-inflammatory activity [16].

The aim of this study is to evaluate the effects of single doses of fucoidan isolated from *E. crinita* on carrageenan-induced paw inflammation in rats and on the serum levels of TNF-α, IL-1β, IL-6, and interleukin 10 (IL-10) of rats with LPS-induced systemic inflammation after 14 days of treatment. Moreover, in the present paper, the antioxidant potential of *E. crinita* fucoidan to scavenge free radicals (DPPH assay) and to reduce ferrous ions by donating an electron (FRAP assay) were evaluated.

## 2. Materials and Methods

### 2.1. Algae Material and Chemicals

*Ericaria crinita* (Duby) Molinari & Guiry talluses were collected from Arapya, Black Sea, Bulgaria (42°11′17.9″ N, 27°50′20.0″ E), in July 2019. The botanical identification and algae mass pre-treatment were described in our previous studies [16]. Fucoidan from *Fucus vesiculosus* (Phaeophyceae) (Product No. F5631, Batch No. SLBC4004V), lipopolysaccharides from *E. coli* O55:B5 (LPS), λ-carrageenan (Product No. 22049, Batch No. BCBP8978V), butylated hydroxytoluene (BHT), ascorbic acid, 2,4,6-tris(2-pyridyl)-s-triazine (TPTZ), and 2,2-diphenyl-1-picryl-hydrazyl-hydrate (DPPH) were purchased from Sigma Aldrich (St. Louis, MO, USA). The solution for the injection of diclofenac sodium (Almiral^®^, Medochemie, Limassol, Cyprus) was purchased from a pharmacy store. All other chemicals and solvents used in this study were of analytical grade. The tested fucoidans, λ-carrageenan, and LPS were dissolved in saline.

### 2.2. Animals

The experiments were performed on male Wistar rats with mean weight of 150–270 g. Animals were housed in standard laboratory conditions: temperature 22 ± 1 °C, humidity 45%, 12:12 h light/dark cycle, and food and water *ad libitum*. 

### 2.3. Extraction Process and Structural Characterization of Fucoidan

The extraction process of *E. crinita* fucoidan (ECF) and its structural characterization were described in our previous study [16]. Briefly, before the extraction, the algae mass was soaked in an ethanol:chloroform:water solution (80:5:15, *v*/*v*/*v*) for depigmentation and delipidation. Then, fucoidan was isolated by acidic extraction using 0.1 M HCl for 2 h at a temperature of 60 °C. The extraction yield of the crude fucoidan was 5.15%, calculated as the percent of the algae dry weight. Neutral sugars (46.64%), uronic acids (13.15%), sulfate groups (17%), total polyphenols (<0.10%), and proteins (0.56%) were estimated colorimetrically. The monosaccharide composition, molecular weight, and structural characteristics of *E. crinita* fucoidan were determined using high-performance anion-exchange chromatography/pulsed amperometric detection (HPAEC-PAD), Fourier-transform infrared (FTIR), size-exclusion chromatography–multi-angle light scattering (SEC/MALS), and proton nuclear magnetic resonance (^1^H NMR) spectroscopy, respectively. Therefore, the ECF was identified as a sulfated xylogalactofucan composed of fucose (39.74%), xylose (20.75%), galactose (15.51%), and glucuronic acid (13.52%), but also small amounts of glucose (5.50%), rhamnose (2.37%), and arabinose (2.13%). ECF consisted of three fractions: high-molecular-weight fraction (Mw = 5.34 × 10^5^), medium-molecular-weight fraction (Mw = 7.01 × 10^4^), and low-molecular-weight fraction (Mw = 1.38 × 10^4^). Furthermore, the complex sulfated heterogeneous structure of the isolated fucoidan was proved by ^1^H NMR analysis [16]. The spectra revealed five characteristic regions which were assigned to the -CH_3_ group (the main characteristic of the fucopyranose unit): α-(1→3)-linked L-fucosyl residues, α-(1→4)-linked L-fucose, H2–H6 protons of sugar residues, the acetyl amine group of the hexose/pentose sugar moiety, or the presence of functional groups such as amino acids, carboxylic acids, or phenols. 

### 2.4. Carrageenan-Induced Paw Edema

Forty male Wistar rats (weight 150–180 g) were allocated randomly into five groups (*n* = 8) and treated intraperitoneally. The rats of the 1st group (control) received 0.9% saline (0.1 mL/100 g bw), animals in in the 2nd group (diclofenac) received diclofenac sodium at a dose of 25 mg/kg bw, and the animals in the 3rd group (fucoidan standard) received 50 mg/kg bw fucoidan from *Fucus vesiculosus*. The experimental rats from the 4th group (fucoidan 25 mg/kg) received 25 mg/kg bw fucoidan from *E. crinita*, and those from the 5th group (fucoidan 50 mg/kg) received 50 mg/kg bw fucoidan from *E. crinita*. The injected volume was 0.1 mL/100 g bw. A subplantar injection of 0.1 mL of a 1% solution of degraded λ-carrageenan in saline was applied 1 h after treatment with the substances into the right hind paw. The paw volume was measured immediately before carrageenan injection and at the 1st, 2nd, 3rd, 4th, and 5th hour after the injection using a Plethysmometer apparatus (Ugo Basile, Gemonio, Italy) as described before [17]. 

Calculation of the paw edema was based on the following formula:

Percentage of increase (%) = [(*Vn* − *V*_0_)/*V*_0_] × 100 (1)

where *Vn* = the volume of the right hind paw measured after carrageenan injection at the *n*-th hour and *V*_0_ = the volume of the right hind paw measured for the same rat before carrageenan injection.

### 2.5. Pro-Inflammatory and Anti-Inflammatory Cytokine Evaluation

Twenty-four male Wistar rats (weight 170–270 g) were allocated into three groups of eight and treated intraperitoneally for 14 days with the following substances: the 1st group (control) received saline injection (0.1 mL/100 g bw); the 2nd group (fucoidan 25 mg/kg) and the 3rd group (fucoidan 50 mg/kg) were treated with 25 mg/kg bw and 50 mg/kg bw fucoidan from *E. crinita*, respectively. The solution of LPS in 0.9% saline (0.25 mg/kg) was injected intraperitoneally thirty minutes after the last application of the substances. Four hours after the LPS challenge, the blood samples were collected in test tubes for serum yield. The latter were transported immediately to the Department of Medical Microbiology and Immunology in an ice container.

In the Medical Microbiology and Immunology Department, the obtained blood samples were promptly subjected to centrifugation at 1000× *g* for a duration of 10 min at room temperature. The resulting supernatant (serum) was carefully aspirated using sterile techniques and divided into smaller portions (aliquots) of 250–500 μL to prevent the need for repeated freeze–thaw cycles, which could affect the stability and quality of samples. All aliquots were placed in labeled cryovials and kept frozen at a temperature of −80 °C until further analysis. The serum concentrations of four cytokines—Rat IL-1β, Rat IL-6, Rat IL-10, and Rat TNF-α—were assessed using a specific enzyme-linked immunosorbent assay (ELISA) with precoated strip plates manufactured by Diaclone (Besancon Cedex, France). These kits were chosen based on their established sensitivity, specificity, and compatibility with rat serum samples. The procedures were carried out in strict accordance with the manufacturer’s provided instructions. Detection of optical density was conducted at 450 nm, with an optional 620 nm reference filter, using a Tecan Sunrise Microplate Reader (Tecan Austria GmbH, Salzburg, Austria) in conjunction with the Magellan™ Data Analysis Standard v7.2 Software. A standard curve was generated using known concentrations of the cytokine standards provided in the ELISA kits. The linear equation derived from this curve was used to calculate the concentrations of cytokines in the samples. The levels of cytokines were presented as picogram per milliliter (pg/mL).

### 2.6. Determination of Antioxidant Activity

#### 2.6.1. 2,2-Diphenyl-1-picryl-hydrazyl-hydrate Radical Scavenging Activity (DPPH)

The DPPH free-radical scavenging activity of *E. crinita* fucoidan was carried out following the protocol of Kao and Chen [18] with some modifications. An amount of 0.5 mM DPPH in methanol (0.2 mL) was added to polysaccharide solutions (1 mL) at different concentrations (0.05–2.5 mg/mL). The mixtures were dropped off at room temperature in a dark place for 15 min. The decrease in the absorbance was measured at a wavelength of 517 nm using a UV-VIS spectrophotometer Evolution 300 (Thermo Fisher Scientific, Waltham, MA, USA). As a blank, the mixture of polysaccharide solution and methanol was used. Antioxidant activity was expressed as inhibition in percentage. The inhibition percent was quantified using the following equation:

(2) $$\%\;\mathrm{Inhibition}=\frac{A_{0}-A_{15}}{A_{0}}\times 100$$

where A_0_ was the absorption of the blank sample at 0 min and A_15_ was the absorption of the polysaccharide solution after 15 min. Data were expressed by triplicate measurements with standard deviation (±SD). The IC_50_ values were calculated from the linear regression plots. 

#### 2.6.2. Ferric Reducing Antioxidant Power (FRAP)

The ferric reducing antioxidant power was examined by the method proposed by Benzie and Strain [19] with slight modifications. The stock solutions included 300 mM acetate buffer (pH 3.6), 156 mg 2,4,6-tripyridil-striazine (TPTZ) dissolved in 50 mL 40 mM HCl, and 270 mg FeCl_3_*6 H_2_O dissolved in 50 mL distilled water. Briefly, the FRAP reagent was prepared by 100 mL acetate buffer, 10 mL FeCl3*6 H2O solution, and 10 mL TPTZ solution. A volume of 0.3 mL of polysaccharide solution (1 mg/mL) was added to 2.7 mL FRAP reagent. The samples were left at 37 °C for 30 min in a dark place. Antioxidant activity was determined as an increase in the absorbance at 593 nm. Trolox was used as a standard solution and the results were expressed as μmol Trolox equivalents per gram of polysaccharide (μM TE/g dw). Values were presented by triplicate measurements with ± SD.

### 2.7. Statistical Analysis 

Statistical analysis was performed using SPSS 17.0. The normal distribution was evaluated with One-sample Kolmogorov–Smirnov test. One-way ANOVA and Bonferroni post hoc test were employed for further analysis. The number of tested animals is given as *n*. The results are presented as mean ± SEM and are considered significant at *p* < 0.05.

## 3. Results

### 3.1. Effect of Fucoidan on Carrageenan-Induced Paw Edema in Rats

**Figure 1:**
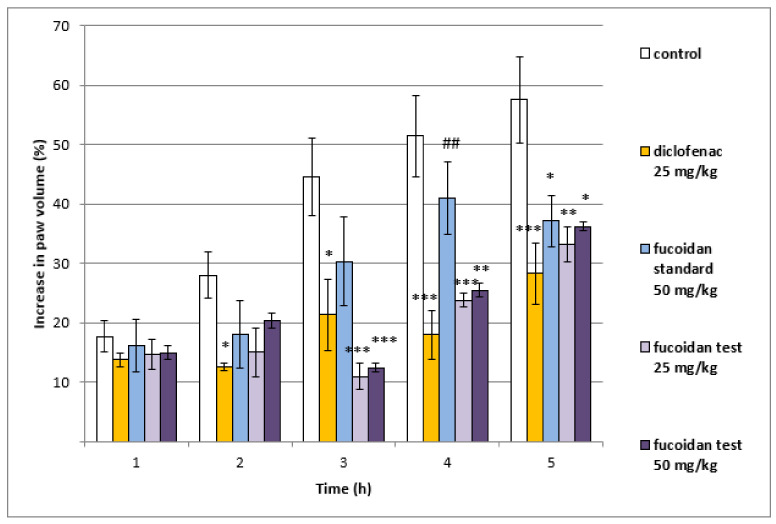
Changes in the paw volume after treatment with diclofenac, fucoidan from *F. vesiculosus* (50 mg/kg bw), and fucoidan from *E. crinita* (25 and 50 mg/kg bw) in a model of inflammation induced by degraded carrageenan in rats: * *p* < 0.05 vs. controls at the same hour; ** *p* < 0.01 vs. controls at the same hour; *** *p* < 0.001 vs. controls at the same hour; ## *p* < 0.01 vs. diclofenac at the same hour.

Fucoidan from *E. crinita* at both doses (25 mg/kg bw and 50 mg/kg bw) shows a well-defined anti-inflammatory effect with peak activity at the 3rd hour after the application (10.93 ± 2.16 for the lower dose and 12.38 ± 0.70 for the higher dose vs. 44.56 ± 6.49; *p* < 0.001) when compared to controls. The effect was also present at the 4th hour for 25 mg/kg bw fucoidan from *E. crinita* (23.81 ± 1.20 vs. 51.48 ± 6.88; *p* < 0.001) and 50 mg/kg bw fucoidan from *E. crinita* (25.51 ± 1.16 vs. 51.48 ± 6.88; *p* < 0.01). At the 5th hour of the experiment, a significant decrease in the paw edema in comparison to control rats was registered after treatment with fucoidan from *E. crinita* at doses of 25 mg/kg bw (33.21 ± 2.98 vs. 57.57 ± 7.23; *p* < 0.01) and 50 mg/kg bw (36.20 ± 0.74 vs. 57.57 ± 7.23; *p* < 0.05). At the same time point, a significant anti-inflammatory effect in the rats treated with fucoidan from *F. vesiculosus* at a dose of 50 mg/kg bw (37.17 ± 4.31 vs. 57.57 ± 7.23; *p* < 0.05) was also observed (Figure 1). 

### 3.2. Alterations in Serum Levels of Pro-Inflammatory Cytokines (TNF-α, IL-1β, and IL-6) 

**Figure 2:**
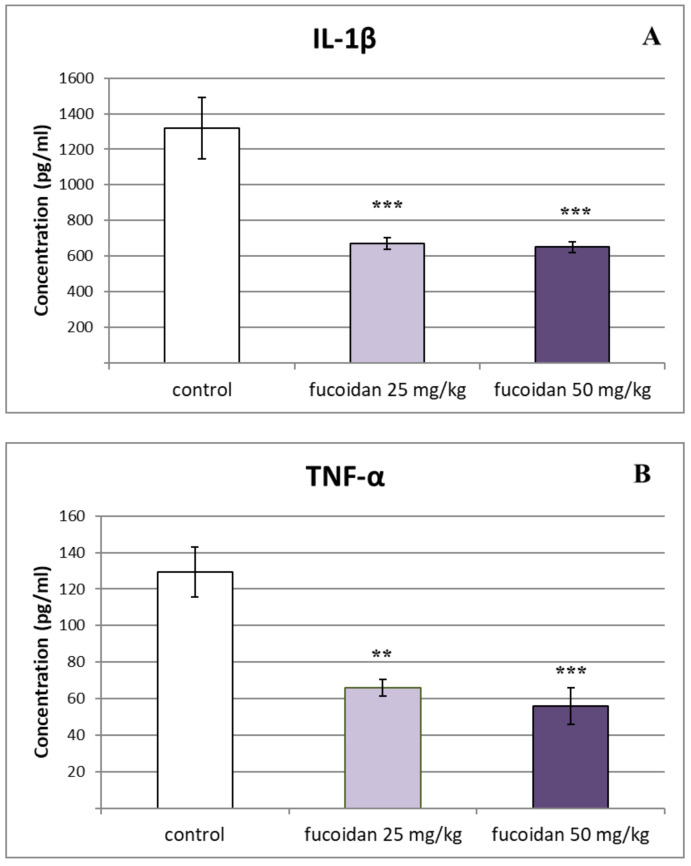

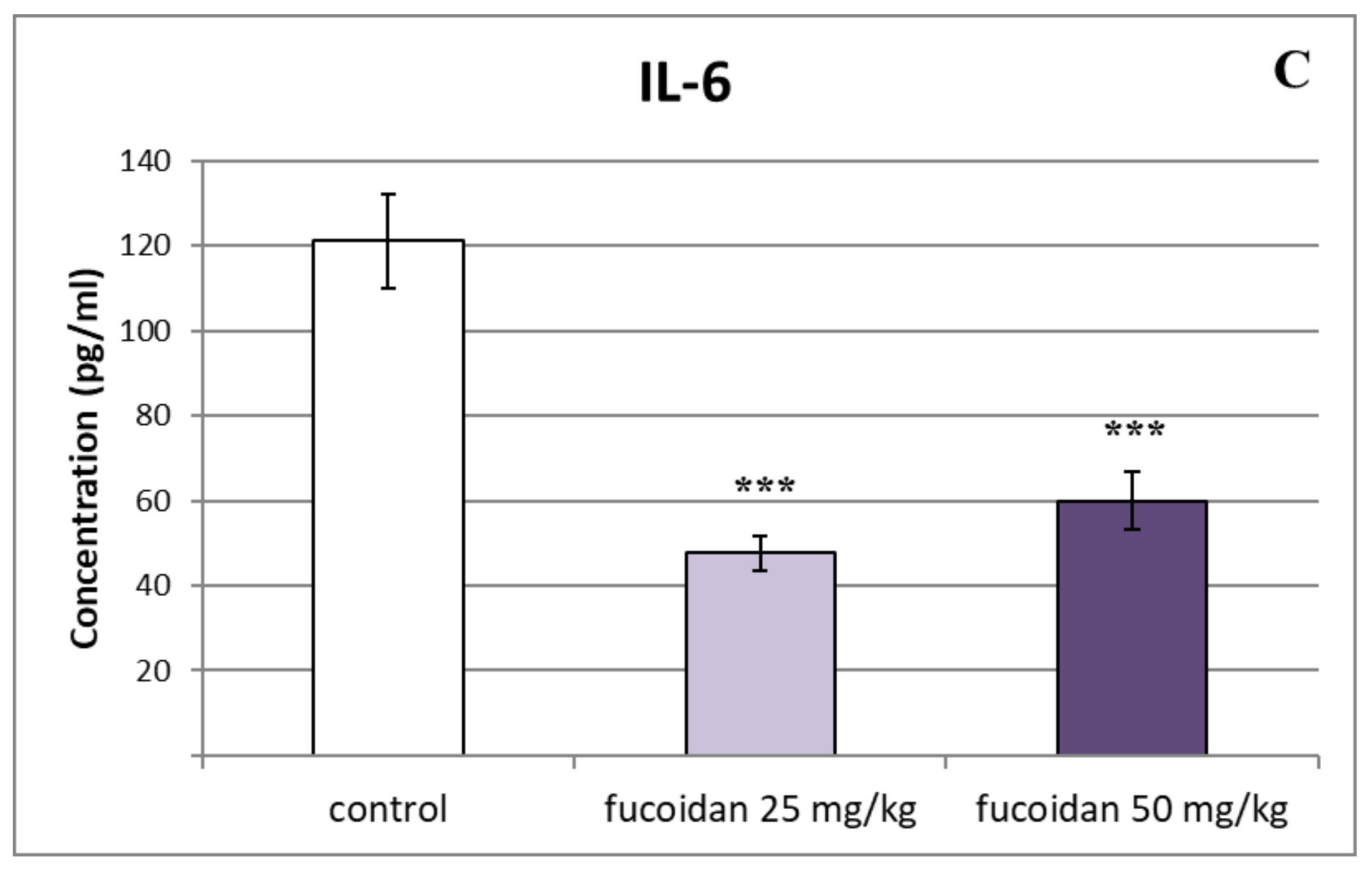
Effect of prolonged treatment with fucoidan from *E. crinita* (25 and 50 mg/kg bw) on serum levels of the pro-inflammatory cytokines IL-1β (panel (**A**)), TNF-α (panel (**B**)), and Il-6 (panel (**C**)) in LPS-induced system inflammation in rats: ** *p* < 0.01 vs. same cytokine controls; *** *p* < 0.001 vs. same cytokine controls.

Subchronic treatment of rats with *E. crinita* fucoidan at a dose of 25 mg/kg bw induced a significant decrease in the serum levels of pro-inflammatory cytokines IL-1β (668.22 ± 32.93 vs. 1318.97 ± 171.14; *p* < 0.001), TNF-α (66.1 ± 4.57 vs. 129.33 ± 13.81; *p* < 0.01), and IL-6 (47.69 ± 4.06 vs. 121.08 ± 11.16; *p* < 0.001) in comparison to saline-treated rats with a model of LPS-induced systemic inflammation. We also observed reduced levels of IL-1β (650.28 ± 30.77 vs. 1318.97 ± 171.14; *p* < 0.001), TNF-α (55.79 ± 9.92 vs. 129.33 ± 13.81; *p* < 0.001), and IL-6 (60 ± 6.7 vs. 121.08 ± 11.16; *p* < 0.001) in rats injected for 14 days with fucoidan at a dose of 50 mg/kg bw when compared to control rats, as shown in Figure 2.

### 3.3. Alterations of Serum Levels of Anti-Inflammatory Cytokine Levels (IL-10) 

**Figure 3:**
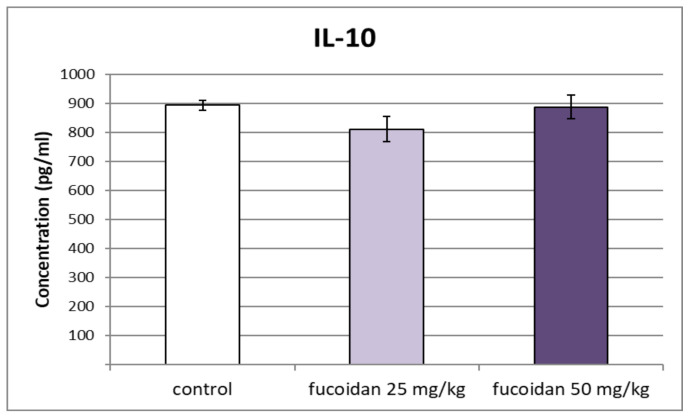
Effect of administration of multiple doses of fucoidan from *E. crinita* (25 and 50 mg/kg bw) on IL-10 serum levels in rats with LPS-induced systemic inflammation.

No significant changes in the levels of the anti-inflammatory cytokine IL-10 were registered after subchronic treatment with *E. crinita* fucoidan at doses of 25 and 50 mg/kg bw in comparison to the group injected with saline (Figure 3).

### 3.4. Antioxidant Activity

#### 3.4.1. DPPH Scavenging Effect

**Figure 4:**
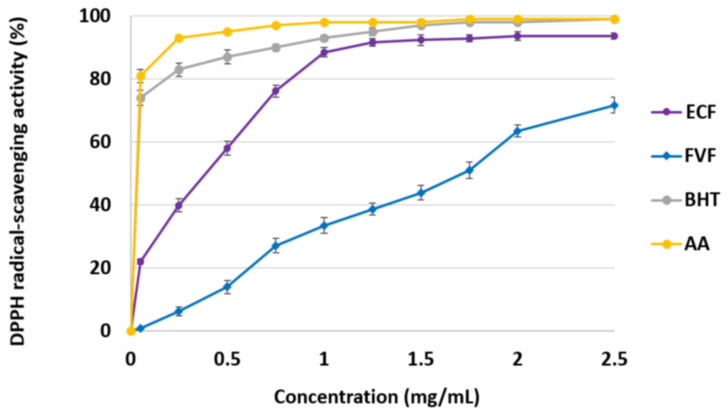
DPPH radical scavenging effect of *E. crinita* fucoidan (ECF), commercial fucoidan from *F. vesiculosus* (FVF), butylated hydroxytoluene (BHT), and ascorbic acid (AA).

The DPPH radical scavenging activity of *E. crinita* fucoidan (ECF) was compared to commercial fucoidan from *F. vesiculosus* (FVF) and two conventional natural antioxidants: butylated hydroxytoluene (BHT) and ascorbic acid (AA). As shown in Figure 4, the inhibition percentage of polysaccharide samples showed a concentration-dependent pattern. At a concentration of 1.25 mg/mL, ECF reached inhibitory activity of 92%, while at the same concentration, FVF exhibited a 39% inhibitory activity. On the other hand, at concentrations of 1.25–2.5 mg/mL, the scavenging effect of ECF remained approximately within the same ranges (91.7–93.6%), while the inhibitory effect of FVF increased from 39% to 72%. Nevertheless, ECF and FVF (IC_50_ = 412 μg/mL and IC_50_ = 1.66 mg/mL, respectively) exhibited lower scavenging activities compared to both BHT (IC_50_ = 5.5 μg/mL) and AA (IC_50_ = 4.1 μg/mL).

#### 3.4.2. Ferric Reducing Antioxidant Power (FRAP)

The antioxidant capacity of *E. crinita* crude fucoidan to reduce the TPTZ–Fe (III) complex to the TPTZ–Fe (II) complex is shown in Table 1. The FRAP value of the crude fucoidan from *E. crinita* (118.72 ± 2.11 μM TE/g) was almost two times higher than the commercial fucoidan from *F. vesiculosus* (75.50 ± 0.93 μM TE/g). However, the synthetic antioxidant BHT still exhibited the highest reducing capacity, 532.65 ± 3.15 μM TE/0.1 mg BHT.

## 4. Discussion

Carrageenan-induced paw inflammation in rats is a model of inflammation that is commonly used in the research of new anti-inflammatory agents. The inflammatory response to this phlogistic agent consists of two phases. During the first phase (1 h after the injection), the inflammation is related to the increased production of histamine, serotonin, and bradykinin, whereas the second phase is governed by the production of neutrophil-derived free radicals, pro-inflammatory cytokines, NO synthesis, neutrophil infiltration, and COX-2 activation, resulting in increased prostaglandin production [20,21]. 

Recently, fucoidan derived from different algal sources has been a subject of intensive research [22,23,24,25]. We registered a well-defined anti-inflammatory activity of fucoidan from *E. crinita* in carrageenan-induced rat paw edema. Similar activity was reported for fucoidan isolated from *Undaria pinnatifida* (Phaeophyceae) [26] and fucoidan from *Turbinaria ornata* (Phaeophyceae) [27]. Fucoidan isolated from seaweeds from the genera *Cystoseira*/*Ericaria* was also found effective in this experimental model. *Ericaria sedoides*, *Cystoseira compressa*, and *Ericaria crinita* fucoidans reduced paw inflammation in this model [28]. Ammar et al. reported a significant decrease in paw edema on the 1st, 3rd, and 5th hour after treatment with *E. crinita* fucoidan at the same doses (25 and 50 mg/kg bw) as in the current study [28]. Our results indicated that *E. crinita* fucoidan alleviated paw edema during the late phase of the inflammation (3rd to 5th hour after degraded carrageenan injection). This result could be related to decreased free-radical production, suppressed production of pro-inflammatory cytokines, NO, or reduced COX-2 activity. 

Due to the major role of the pro-inflammatory cytokines in the late phase of inflammation, we examined the serum levels of TNF-α, IL-1β, and IL-6 in rats with systemic inflammation. Previously, we reported decreased levels of TNF-α and IL-1β after a single dose of fucoidan in the same animal model [16]. Our current research revealed that continuous application of fucoidan evokes more prominent changes in cytokine levels. These results are supported partially by the in vitro findings of Lee et al., Ni et al., and Fernando et al. [1,22,29]. These authors also reported such decrease after in vitro treatment of LPS-stimulated RAW 264.7 macrophages with fucoidan fractions derived from *Ecklonia cava, Saccharina japonica*, and *Chnoospora minima* (Phaeophyceae). Similar activity and decreased levels of TNF-α, IL-1β, and IL-6 in serum were reported for fucoidan isolated from *Saccharina japonica* in a model of streptozotocin-induced diabetes mellitus in rats after 4 weeks of treatment [30]. Fucoidan from the same algal source (*Saccharina japonica*, also known as *Laminaria japonica*) was tested in mice with a model of liver damage, and the results revealed the anti-inflammatory effect of fucoidan [31]. Prolonged fucoidan treatment (21 days) with doses of 50 or 100 mg/kg/day evoked a significant decrease in the serum levels of TNF-α, IL-1β, and IL-6 when compared to animals treated only with the hepatotoxic agent. 

Our results could not exclude the involvement of other factors in the anti-inflammatory activity of the isolated fucoidan. This effect could also be related to attenuated COX activity. Even though we did not measure the COX activity, some research articles report decreased COX-2 activity *in vitro*. *Turbinaria decurrens* (Phaeophyceae) fucoidan was found effective in formalin-induced paw edema in mice, as reported by Manikandan et al. [23]. The authors registered decreased expression of COX-2 genes [23].

Regarding the levels of the anti-inflammatory cytokine IL-10, our results showed no significant changes in its serum levels after treatment with fucoidan. In contrast, *Saccharina japonica* (formerly *Laminaria japonica*) (Phaeophyceae) fucoidan significantly increased the level of IL-10 in serum in rats with a model of myocardial infarction [32]. An elevated level of this cytokine after in vitro treatment of the Caco-2 cell line with low-molecular-weight fucoidan from *Sargassum hemiphyllum* was reported by Hwang et al. [33]. The observed differences could be explained by the different algal sources, as reported by Matsumoto et al. [34]. Fucoidan derived from *Cladosiphon okamuranus* Tokida decreased IFN-γ and IL-6 synthesis and increased levels of IL-10 in lamina propria of the colon, while no changes in the levels of these cytokines were observed after treatment with fucoidan from *Fucus vesiculosus* [34].

The evaluation of the antioxidant potential of marine algae polysaccharides, the elucidation of their mechanism, and the factors influencing their antioxidant activity have been a subject of many recent studies [7,15,35]. Fucoidan is known as a scavenger of free radicals, superoxide, and hydroxyl radicals, as well as a chelator and reductor of iron ions [36,37]. The antioxidant activity of fucoidan can be influenced by several factors, including concentration, molecular weight, monosaccharide composition, glycosidic linkages, degree of sulfation, and substituents in the molecule and their position [38]. High-molecular-weight fucoidans (HMWF) hardly pass through the phospholipid bilayer and exhibit a lower effect, while medium-molecular-weight fucoidans (MMWF) and low-molecular-weight fucoidans (LMWF) exhibit stronger antioxidant activity [39]. However, other authors reported that the relationship between the molecular weight of fucoidans and their antioxidant activity is not linear [40]. The presence of sulfate groups increases the antioxidant effect, while cationic groups, for example, amino groups, have little effect on antioxidant activity because they cannot activate hydrogen atoms [36,41]. The extraction method of the polysaccharide also affects the antioxidant activity, as it depends on the content and location of sulfate groups in the polymer chain [42].

The crude fucoidan isolated from *E. crinita* (ECF) demonstrated DPPH and FRAP antioxidant activity. As determined by the DDPH assay, the IC_50_ values of ECF (412 μg/mL) were close to those reported for fucoidan isolated from Tunisian *Cystoseira compressa* (IC_50_ = 430 μg/mL), with sulfate and fucose contents of 14.65% and 62.46%, respectively [15]. Fucoidan isolated from *Ericaria crinita* reached the highest inhibitory effect of 91.7–93.6% at concentrations of 1.25–2.5 mg/mL, while *C. compressa* fucoidan displayed a 90% inhibitory effect at a concentration of 1.5 mg/mL. Moreover, Sellimi et al. [35] reported a 100% DPPH inhibition activity for *Gongolaria barbata* (formerly *Cystoseira barbata*) (Phaeophyceae) fucoidan (sulfates 22.5%, fucose 44.6%) at a concentration of 1.5 mg/mL. Ammar et al. [43] reported a DPPH free radical scavenging activity of 77% at a concentration of 5 mg/mL for fucoidan extracted from *Ericaria sedoides* with an Mw of 140,000 g/mL, a sulfate content of 15.5%, and a fucose content of 17.6%. The higher scavenging effect of *Gongolaria barbata* fucoidan compared to *Ericaria crinita*, *Cystoseira compessa*, and *Ericaria sedoides* fucoidan could be related to its higher content of sulfate groups. Fucoidans isolated from *Cystoseira*/*Eicaria*/*Gongolaria* species resulted to be more effective radical scavenging agents compared to other brown algae such as *Ascophyllum nodosum* (43.0%, IC_50_ = 1.16 mg/mL) [44], *Undaria pinnatifida* (86.80% at concentration 1 mg/mL) [45], *Sargassum pallidum* (19.1%), *S. vulgare* (22.5%), and *S. tenerrimum* (64.66%) at concentrations of 2.5 mg/mL, 3.8 mg/mL, and 1 mg/mL, respectively [15]. Compared to crude fucoidans, the established lower scavenging effect of the commercial fucoidan from *F. vesiculosus* (IC_50_ = 1.66 mg/mL), also reported by other authors (IC_50_ = 2.30 mg/mL [44]; 76.61% at 4 mg/mL [46]), could be related to its higher purity and the absence of residual substances such as phenols, pigments, and proteins. 

The crude fucoidan from *E. crinita* displayed the potential to reduce the Fe^3+^/ferricyanide complex at concentrations of 1 mg/mL (Table 1). Several studies on the FRAP capacity of fucoidan isolated from *Cystoseira*/*Eicaria*/*Gongolaria* species have been conducted [15,35,43,44]. However, the application of different variations of the FRAP method and the use of different standards, equivalents, and units complicate the comparison of the antioxidant values. For *C. compressa* fucoidan, Hentati et al. [15] reported a significant FRAP activity with an OD_700_ = 0.75 at a concentration of 0.5 mg/mL. At a concentration of 10 mg/mL, the Tunisian *Ericaria sedoides* fucoidan exerted a reducing activity with an OD_700_ = 1.6 [43]. Moreover, Sellimi et al. [35] observed the highest reducing activity of *Gongolaria barbata* fucoidan at concentrations of 1 mg/mL and 24.62 mg ascorbic acid equivalents/g of the sample. Correlating with our results, Rajauria et al. [44] reported FRAP values for *A. nodosum* crude fucoidan and commercial fucoidan from *F. vesiculosus* to be 38.6 and 13.9 mg TE/g, respectively. The reducing capacities of fucoidans are supposed to be due not only to their sulfate content and molecular weight but also to the presence of hydroxyl and carboxylic groups of the uronic acids in their chain [15].

## 5. Conclusions

Fucoidan from *Ericaria crinita* showed a well-defined anti-inflammatory effect during the late phase of the carrageenan-induced rat paw edema. The subchronic application of this sulfated polysaccharide at doses of 25 and 50 mg/kg bw significantly decreased the levels of all tested pro-inflammatory cytokines (IL-1β, TNF-α, and Il-6) in the serum of rats with a model of LPS-induced system inflammation, while the anti-inflammatory cytokine IL-10 was not affected. The performed in vitro DPPH and FRAP analyses indicated the potent antioxidant activity of the isolated polysaccharide—IC_50_ = 412 µg/mL and 118.72 μM Trolox equivalent/g. The reported results reveal the potential of *E. crinita* fucoidan as an anti-inflammatory agent and the ability of the polysaccharide to effectively reduce oxidation and prevent oxidative stress.

## Figures and Tables

**Table 1:** Ferric reducing antioxidant power of *E. crinita* fucoidan (ECF), commercial fucoidan from *F. vesiculosus* (FVF), and butylated hydroxytoluene (BHT).

Sample	Concentration	FRAP μM TE/g
ECF	1 mg/mL	118.72 ± 2.11
FVF	1 mg/mL	75.50 ± 0.93
BHT	0.1 mg/mL	532.65 ± 3.15

## Data Availability

The data presented in this study are available on request from the corresponding author.
